# Differences in healthcare access between Rohingya refugees and their host community in Cox's Bazar, Bangladesh

**DOI:** 10.3389/fpubh.2025.1712128

**Published:** 2025-12-11

**Authors:** Samaha Masroor Saqib, Shirin Ziaei, Soorej Jose Puthoopparambil

**Affiliations:** 1 Department of Women's and Children's Health, Uppsala University, Uppsala, Sweden; 2 Department of Food Studies, Nutrition and Dietetics, Uppsala University, Uppsala, Sweden

**Keywords:** Rohingya refugees, host community, healthcare access, Bangladesh, health inequalities

## Abstract

**Background:**

Forced migration is increasing globally and majority of the forced migrants are displaced in low- and middle-income countries. Bangladesh, which hosts about one million Rohingya refugees in one of the world's largest refugee camps in Cox's Bazar, faces challenges in ensuring equitable healthcare access for both refugees and host communities. This can create disparities in healthcare access for both the groups. Understanding the disparities is critical to aid in effective healthcare strategies that address the needs of both groups and promote social cohesion. This study investigated the differences in healthcare access between Rohingyas and their host community in Cox's Bazar, Bangladesh.

**Methods:**

Secondary data, covering refugee and host households using two datasets: household-level and individual-level were analyzed. Generalized linear mixed model and binary logistic regression was used to explore healthcare access differences between Rohingya refugees and their host community.

**Results:**

Refugees were significantly more likely to face financial barriers (OR = 3.10, 95% CI: 2.14, 4.49; *p* = <0.001) when accessing healthcare compared to their host community. However, refugees had higher odds of enrolling in antenatal care (OR = 2.26, 95% CI: 1.03, 4.65; *p* = 0.026) and nutrition programs (OR = 20.99, 95% CI: 10.64, 41.41; *p* = <0.001). Additionally, they were more likely to receive clear COVID-19 awareness information (OR = 1.75, 95% CI: 1.17, 2.63; *p* = 0.006) and access proper healthcare (OR = 3.10, 95% CI: 2.19, 4.39; *p* = <0.001) compared to their host community.

**Conclusion:**

The findings indicate that both refugees and host face healthcare access barriers. The refugee may benefit from targeted interventions in camps, leading to better access to specific healthcare services. To reduce disparities, a more inclusive healthcare strategy is necessary, ensuring equitable access for both refugees and host populations.

## 1 Introduction

Over the past decade, the number of forced migrants has surged, reaching a record high of 117.3 million in 2023, with 75% of the refugees (and other people in need of international protection) hosted in low- and middle-income countries (LMICs) that face several resource constraints including constrains related to health system (1–3). Refugees are the largest group of internationally displaced forced migrants (1). A refugee is defined as a “person who owing to a well-founded fear of being persecuted for reasons of race, religion, nationality, membership of a particular social group or political opinion, is outside the country of their nationality or habitual residence and is not able to, or because of such fear, is not willing to avail themselves of the protection of that country” (4).

Right to the highest attainable standard of health is a fundamental human right for everyone, irrespective of nationality or legal status (5). Forced migration, being a vital factor affecting health, can lead to poor health outcomes for forced migrants resulting in disparities in health outcomes between them and their host community (6, 7).

Access to healthcare is a complex concept that encompasses several critical dimensions, including approachability, acceptability, availability, affordability, and appropriateness of health services (6). Ensuring access to comprehensive healthcare is crucial for safeguarding and promoting public health, as well as achieving health equity for all individuals. Furthermore, healthcare access plays a significant role in fostering social cohesion, particularly in communities that include both host populations and forced migrants, contributing to a more integrated society (8).

While generally host countries welcome refugees, they might be unprepared to accommodate a large number of refugees, especially in LMIC contexts. This is because forced displacement brings numerous challenges that require sudden, significant adjustments and reorientation such as socio-cultural, economic, environmental and sharing of resources (9, 10). Due to the physical and psychological trauma that refugees may endure in their home countries, during their journey, and throughout the resettlement process, they often experience poorer health compared to long-established residents (hosts) and are in need of healthcare (7, 11, 12). Moreover, access to healthcare for refugees is frequently hindered by numerous obstacles, such as language barrier, cultural differences, inadequate information about available services, and lack of financial resources, unlike the host population, who are often known to have better access to healthcare services (13, 14). On the other hand, the host communities in LMIC contexts are grappling with significant challenges such as limited access to healthcare, financial instability and inadequate infrastructure, even before the arrival of the refugees. The arrival of refugees, often suddenly and in relatively large numbers, exacerbates these existing issues, putting additional strain on already stretched resources (14).

Policies on reception of refugees, including healthcare, differ widely with some countries providing comprehensive services, while others impose restrictive measures or deny access to certain services. For example, Uganda, a signatory of the 1951 Refugee Convention and later 1967 Protocol, has incorporated refugee rights into national law with its 2006 Refugees Act and 2010 Refugees Regulations. The Health Sector Integrated Refugee Response Plan (2019–2024) offers service provision that supports refugees within the hosting community (15). While Uganda's inclusive approach is often praised as a model for refugee integration (16), it also places considerable pressure on limited national resources, as healthcare systems have to accommodate a growing population with competing needs. This resource sharing can lead to straining service quality and sustainability (17). On the other hand, Bangladesh is not a signatory to the 1951 Refugee Convention or the 1967 Protocol. As a result, the government recognizes the Rohingyas (refugees from Myanmar) primarily as “Forcibly Displaced Myanmar Nationals,” rather than as formal refugees (18, 19). The lack of recognition as refugees could exacerbate their vulnerability, as it limits their access to broader social services, including public health services, forcing many to rely on overstretched healthcare services provided by non-governmental organizations (NGOs) (20). While humanitarian organizations play a crucial role in delivering medical aid in these underserved areas, the parallel nature of these service structures limits integration and long-term sustainability (21, 22). Similar challenges have been observed in other Asian countries such as Malaysia and Pakistan, where refugees lack formal recognition and depend primarily on informal or NGO-supported healthcare (23, 24). Integrated approaches tend to promote shared access but face challenges of resource strain, while restrictive approaches risk reinforcing disparities and long-term dependency on humanitarian aid (25).

While substantial evidence highlights the barriers to healthcare access that only refugees face (26–28), there is limited research examining how healthcare access may differ between refugees and host communities. Some studies have revealed that refugees, often excluded from health insurance programs, experience greater unmet healthcare needs compared to host populations (29, 30). On the other hand, as stated above, host population may also feel neglected in terms of access to certain services. A study by Weiss et al. (31) showed that overall, more refugees utilized outpatient services compared to the host nationals in several African nations. The allocation of resources to support refugees can lead to perceptions of reduced availability for local residents, increased demand on public services, and economic strain (32, 33). Aid organizations, which focus primarily on emergency relief, may not always include the affected host communities (34). For example, many refugee response plans are primarily designed to address the needs of refugees (35, 36), leaving some of the members of the host community feeling disadvantaged as they were not included in relief programs by both local and international humanitarian agencies (37–39).

Bangladesh has been hosting the Rohingyas (forcibly displaced from Myanmar) for several decades. The first group arrived in 1978, with the largest influx occurring in 2017 when around 700,000 Rohingya sought refuge in Bangladesh. The Rohingyas are currently spread across 34 different camps, primarily in Teknaf and Ukhiya subdistricts in Cox's Bazar (12).

Cox's Bazar is one of the most impoverished regions in Bangladesh, with high poverty rates, inadequate healthcare infrastructure, and economic instability (40, 41). It is located in southeastern Bangladesh and hosts one of the world's largest refugee camps, with Rohingya refugees who fled persecution in Myanmar. The district suffered from limited resources and a weak public health system even before the arrival of the Rohingyas (41). The sudden arrival of a large number of Rohingya refugees exacerbated the existing challenges, placing an overwhelming burden on the healthcare system (42). For example, in 2018, the host community members faced a fifty percent increase in waiting times for healthcare services (19). This shift has led to growing frustrations among host community members, who often perceive that their needs have been deprioritized (37).

Perceptions of neglect and inequity may foster resentment and tension between refugees and host community members, potentially negatively affecting social cohesion (43, 44). The rapid influx of Rohingya refugees in Bangladesh has increased the demand for healthcare services, with humanitarian agencies primarily focusing on refugee healthcare needs (37, 45). The strain on health systems can compromise the ability of equitable distribution of services between the refugees and hosts (39, 46). A study in Bangladesh reported that even when aid was available for the Rohingya refugees over a long period, the poorer segments of the host population received insufficient attention (33), even though under 2018 Joint Response Plan, 25% of the humanitarian aid is to be allocated to support host communities impacted by the influx of refugees (47). Understanding the disparities in healthcare access between the Rohingya refugees and the host community is thus crucial for developing inclusive measures to address the health needs of both the host population and refugees. The aim of the study was to identify whether differences in healthcare access exists between Rohingya refugees and the host community, and the factors associated with it.

## 2 Methods

### 2.1 Data source

This study utilized a cross-sectional design, conducting secondary analyses of publicly available data from the United Nations High Commissioner for Refugees' (UNHCR) “Joint Multi-Sector Needs Assessment: Cox's Bazar, Rohingya Refugee Response—August 2020” survey. Data collection took place between July 28 and August 13, 2020. Approximately 400 households per Upazila (subdistrict), Teknaf and Ukhiya, were selected for interviews, covering both Rohingya refugees and host communities. Sample sizes for each camp were determined using UNHCR's latest population data, with sampling points randomly drawn from the UNHCR refugee registration database, including additional buffer points to account for non-eligibility or non-response. Further methodological details are available elsewhere (48).

Two structured questionnaires were administered in the survey, one at household (HH) level and another at individual level. The HH survey employed the same questionnaire for both refugee HHs and host HHs, and the individual survey employed the same questionnaire for individuals from both the groups. The HH dataset consists of 1,747 records (836 refugee HHs, 911 host HHs), while the individual dataset consists of 9,338 records (4,292 refugees and 5,046 hosts individuals) (48).

Because of the movement restrictions due to the preventative measures for COVID-19 during data collection and with limited access to camps, all interviews were conducted over the phone. Since phone ownership was more common among men, female-headed HHs were sampled proportionately to their representation in the database to ensure adequate representation of women (48).

### 2.2 Variables

Four separate outcomes [financial barrier, enrollment in antenatal care (ANC) program, enrollment in nutritional program, received clear COVID-19 awareness information] were studied at the HH level, and a fifth outcome (accessing proper healthcare) at the individual level.

#### 2.2.1 Household survey (Outcomes 1–4)

##### 2.2.1.1 Exposure

The exposure variable was the migratory status of HH, categorized as refugee HH versus host HH.

##### 2.2.1.2 Outcome

Healthcare access as an outcome, is a complex and multi-dimensional concept. Healthcare access as a composite measure was not directly assessed as an outcome variable. Instead, separate outcome variables available in the dataset were chosen that pertained to healthcare access. These included whether the HH faced financial barriers to accessing healthcare (Outcome 1), whether pregnant women in the HH were enrolled in the ante-natal care (ANC) program (Outcome 2), whether pregnant and/or lactating women (PLW) in the HH were enrolled in the Nutrition Program (Outcome 3), and whether the HH received clear information about accessing healthcare during the COVID-19 pandemic (Outcome 4).

Financial barrier to healthcare access (Outcome 1) was categorized by determining whether HHs experienced financial barriers while accessing healthcare when a member of the HH was seriously ill or died in the past 30 days. Specifically, this was assessed based on whether the HH had to go into debt to pay for health expenditures, seek community support to pay for services, opt for lower-quality healthcare/cheaper medication, or resort to home treatment due to lack of money to go to hospital/clinic. Financial barriers were considered to be present if at least one of these four situations occurred and was recorded as “yes”. “No” was recorded for the HH where none of the above-mentioned situations had occurred.

The enrollment of pregnant women in the ANC program (Outcome 2) was also a categorical variable and was defined by asking whether there were pregnant women in the HH and if they were currently enrolled in an ANC program. Enrollment was recorded as “yes” if at least one pregnant woman in the HH was enrolled in the ANC program, and “no” if none were enrolled.

The enrollment of PLW in a nutrition program (Outcome 3) was measured by asking if there were any PLW in the HH and whether they were enrolled in a specific nutrition program. The outcome was categorized as “yes” if at least one PLW was enrolled, and “no” if none of the PLW were enrolled.

For the variable assessing whether HHs received clear COVID-19 awareness information (Outcome 4), respondents were asked if they had received information on where to go or whom to contact if they had symptoms of COVID-19. The response was categorized as “yes” if the HH confirmed receiving such information and “no” if they did not.

##### 2.2.1.3 Covariates

The initial selection of covariates encompassed various sociodemographic and household characteristics. Sex of the head of the HH was a categorical variable with categories male and female. Education level represented the highest attained education of the HH and was categorized into “Higher” (secondary, tertiary, or vocational education) and “Lower” (no formal education, primary education, and madrassa). Household size, defined by the total number of individuals in the HH, was categorized into “Small” (if 1–5 members in HH) and “Large” (if ≥6 members in HH). Lastly, the presence of a disabled person in the HH was a binary categorical variable (present or absent), indicating whether any member of the HH had a disability, which included difficulties in seeing, hearing, walking, remembering/concentrating, self-care, or speaking/communicating. The variables marital status and age of the head of HH were identified as unreliable after conducting preliminary data checks, and therefore excluded from the final analysis.

Same covariates were used with respect to all the four outcome variables.

#### 2.2.2 Individual survey (Outcome 5)

##### 2.2.2.1 Exposure

The exposure variable was the individual's migratory status, categorized as refugee and host.

##### 2.2.2.2 Outcome

Access to proper healthcare (Outcome 5) was a categorical variable created to indicate whether an individual accessed a formal or informal healthcare facility (49, 50) when seeking treatment (Yes or No) during the past 4 weeks. Access to Proper healthcare was defined as accessing services from NGO clinics, government clinics, private clinics, or consulting doctors remotely (formal healthcare facilities/providers). Conversely, seeking treatment at pharmacies, drug shops, traditional healers, or other informal providers (informal healthcare facilities/providers) was categorized as not accessing proper healthcare.

##### 2.2.2.3 Covariates

Age, sex and employment from the individual dataset were the covariates for analysis. Sex was a categorized as male and female, while age of the individual was a continuous variable. Employment status, defined by whether the person worked to earn an income during the past 30 days, was categorized as employed (Yes) or unemployed (No).

### 2.3 Statistical analysis

To understand the characteristics of the data, a descriptive analysis was conducted, where continuous variable was delineated as mean with standard deviation (SD) and categorical variables as frequencies and percentages. Sample weights were used to ensure an accurate representation.

We conducted separate analyses at the HH and individual levels to examine differences in healthcare access between refugees and host communities. Generalized linear mixed model (GLMM) with a logit link and binomial family was used to analyze the HH level data. The GLMM included a random intercept at the sub-district level to adjust for clustering arising from HHs being nested within the two sub-districts (Teknaf and Ukhiya), thereby accounting for potential intra-cluster correlation. The model was further adjusted for relevant HH demographic and socioeconomic characteristics. For the individual level analysis, we applied a binary logistic regression, adjusting for individual sociodemographic characteristics.

Both the models were adjusted for sample weights. Missing data were handled as missing completely at random. All the statistical analyses were carried out using IBM SPSS Statistics 28. A *p*-value <0.05 was considered statistically significant.

### 2.4 Ethical consideration

This study did not require ethical approval since it utilized publicly available anonymized survey data sourced from the UNHCR microdata library and the authors did not have access to the identity of the survey participants. The data is publicly accessible as the likelihood of identifying individual respondents is deemed minimal (51). All UN organizations have an annual blanket approval for data collection from Refugee Relief and Repatriation Commissioner (RRRC) and requires that the organizations keep the RRRC updated about their activities to maintain all necessary standards, including ethical standards (52). The data collection was conducted in coordination with RRRC and the Inter Sector Coordination Group (ISCG) (53).

## 3 Results

### 3.1 Participants

**Figure 1:**
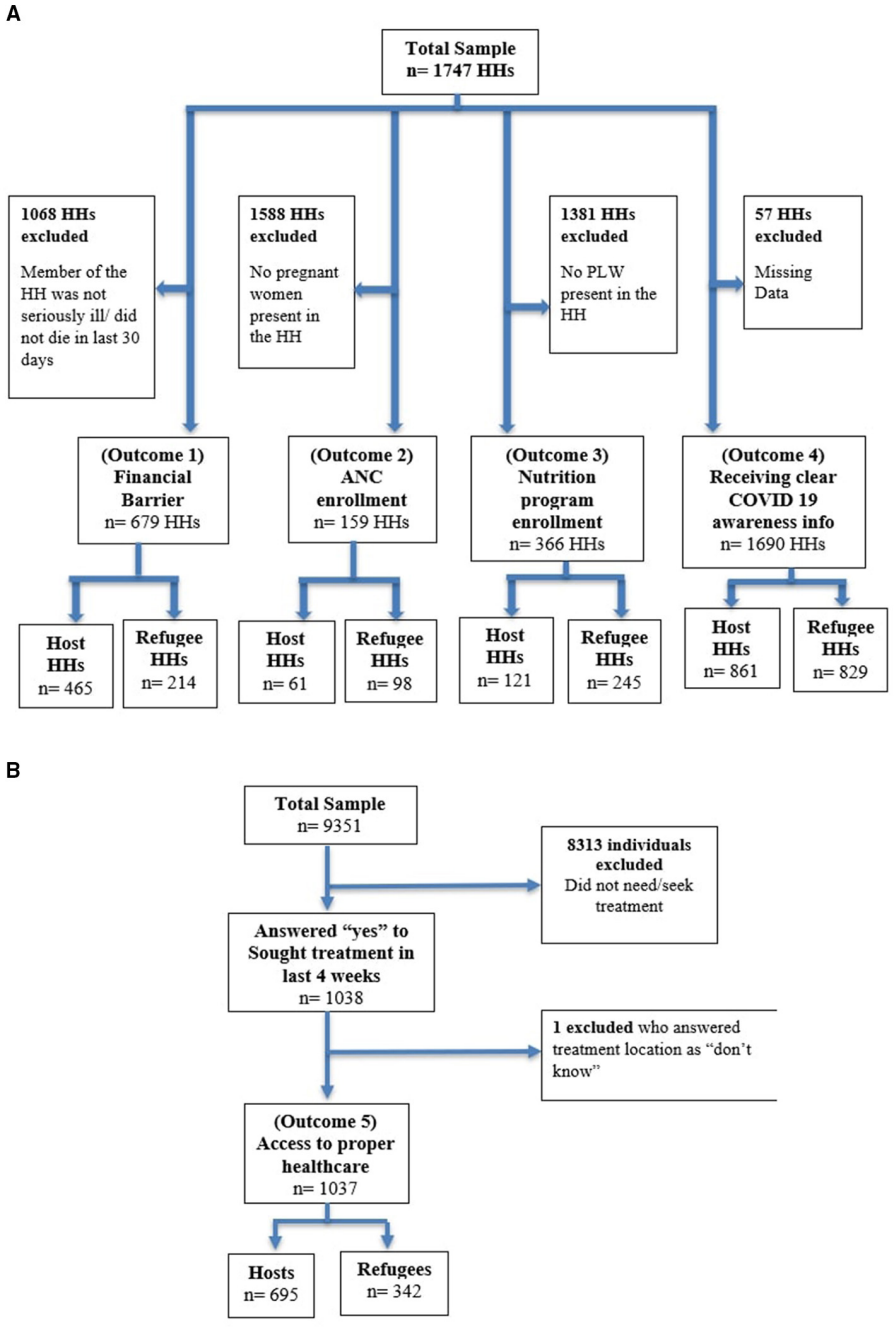
**(A)** Sample selection from HH data based on different outcome variables (weighted). **(B)** Sample selection from individual data (weighted).

In total, the HH survey included 1747 HHs. After exclusion, 679 HHs, 159 HHs, 366 HHs and 1690 HHs were selected for analyzing the difference in financial barriers (Outcome 1), ANC enrollment (Outcome 2), nutrition program enrollment (Outcome 3), and receiving clear COVID-19 awareness information (Outcome 4), respectively (see Figure 1A). From the individual survey data, out of an initial 9351 individuals, 1037 individuals were selected for analyzing the difference in access to proper healthcare (Outcome 5) (see Figure 1B). All the above values are weighted estimates.

### 3.2 Background characteristics of study population

A comparative analysis of (weighted) background characteristics linked to healthcare access indicators based on both HH data and individual data is presented in Table 1. Refugee HHs reported more financial barriers (45.3%) than host HHs (20.2%) but showed higher proportions of ANC enrollment (51.0% vs. 31.1%) and PLW enrollment in nutrition programs (69.4% vs. 11.5%). Additionally, 95.9% of refugee HHs received clear COVID-19 awareness information, compared to 91.4% of host HHs. Female-headed HHs experienced more financial barriers (35.0%) than male-headed HHs (26.7%) and had similar ANC program enrollment proportions (42.3% vs. 43.2%) but higher PLW nutrition program enrollment proportions (53.8% vs. 49.7%). HHs with lower education faced more financial barriers (34.6%) than HHs with higher education (23.1%) but showed higher ANC (45.9% vs. 39.7%) and nutrition program enrollment (58.3% vs. 40.5%). For disability status, HHs without disabled persons encountered more financial barriers (29.9%) but had slightly higher enrollment in ANC (45.1%), nutritional programs (53.8%) and access to COVID-19 awareness info (94.5%) compared HHs with disabled person (25.9%, 41.1%, 42.3%, and 91.6% respectively). Lastly, small HHs reported slightly lower financial barriers (27.8% vs. 28.5%) and greater program enrollments (ANC: 47.9% and nutritional: 51.9%) than larger HHs (ANC: 37.1% and nutritional: 48.4%).

**Table 1:** Background characteristics of households and individuals in relation to outcome variables.

**Characteristics**			**Migratory status**		**Sex**		**Education**		**Disabled person**		**Household size**		**Employed**	
			**Host**	**Refugee**	**Female**	**Male**	**Lower**	**Higher**	**Absent**	**Present**	**Small**	**Large**	**No**	**Yes**
**BASED ON HH DATA**														
Financial barrier (Outcome 1)	n=679 (%)	Yes	94 (20.2)	97 (45.3)	42 (35.0)	49 (26.7)	100^*^ (34.6)	89^*^ (23.1)	155 (29.9)	76 (25.9)	98 (27.8)	93 (28.5)	N/A	N/A
		No	371 (79.8)	117 (54.7)	78 (65.0)	410 (73.3)	189^*^ (65.4)	297^*^ (76.9)	270 (70.1)	218 (74.1)	255 (72.2)	233 (71.5)	N/A	N/A
Enrollment in ante-natal care program *^(^*Outcome 2)	n=159 (%)	Yes	19 (31.1)	50 (51.0)	11^*^ (42.3)	57^*^ (43.2)	39^*^ (45.9)	29^*^ (39.7)	46^*^ (45.1)	23^*^ (41.1)	46^*^ (47.9)	23^*^ (37.1)	N/A	N/A
		No	42 (68.9)	48 (49.0)	15^*^ (57.7)	75^*^ (56.8)	46^*^ (54.1)	44^*^ (60.3)	56^*^ (54.9)	33^*^ (58.9)	50^*^ (52.1)	39^*^ (62.9)	N/A	N/A
Enrollment in nutrition program *^(^*Outcome 3)	n=366 (%)	Yes	14 (11.5)	170 (69.4)	28 (53.8)	156 (49.7)	120^*^ (58.3)	64^*^ (40.5)	141 (53.8)	44 (42.3)	110^*^ (51.9)	74^*^ (48.4)	N/A	N/A
		No	107 (88.4)	75 (30.6)	24 (46.2)	158 (50.3)	86^*^ (41.7)	94^*^ (59.5)	121 (46.2)	60 (57.7)	102^*^ (48.1)	79^*^ (51.6)	N/A	N/A
Receiving Covid-19 awareness info *^(^*Outcome 4)	n=1690 (%)	Yes	787 (91.4)	795 (95.9)	330 (95.1)	1252 (93.2)	780^*^ (94.0)	793^*^ (93.3)	1110^*^ (94.5)	469^*^ (91.6)	920^*^ (94.0)	662^*^ (93.2)	N/A	N/A
		No	74 (8.6)	34 (4.1)	17 (4.9)	91 (6.8)	50^*^ (6.7)	57^*^ (6.7)	64^*^ (5.5)	43^*^ (8.4)	59^*^ (6.0)	48^*^ (6.8)	N/A	N/A
**BASED ON INDIVIDUAL DATA**														
Proper Healthcare *^(^*Outcome 5)	n=1037 (%)	Yes	368 (53.0)	269 (78.7)	350 (62.8)	287 (59.8)	N/A	N/A	N/A	N/A	N/A	N/A	421^*^ (64.0)	52^*^ (57.8)
		No	327 (47.0)	73 (21.3)	207 (37.2)	193 (40.2)	N/A	N/A	N/A	N/A	N/A	N/A	237^*^ (36.0)	38^*^ (42.2)

^*^The total sample size varied due to missing values.

Based on individual data, among refugees, 78.7% reported accessing proper healthcare, compared to 53.0% of the host. A slightly higher proportion of females accessed proper healthcare (62.8%) than males (59.8%). Additionally, higher proportion of unemployed individuals accessed proper healthcare (64.0%) compared to those employed (57.8%). With regards to age distribution (data not shown in the tables) it was seen that individuals accessing proper healthcare (mean 28.4 years ± 22.7 SD) were slightly older compared to individuals not accessing proper healthcare (mean 27.1 years ± 22.9 SD).

### 3.3 Healthcare access at both household and individual levels

The crude (COR) and adjusted odds ratios (AOR) for various outcome variables, with the migratory status of HH/individual (host vs. refugee) as the independent variable is presented in Table 2. For all the analyses, the effect remained significant even after adjusting for covariates.

**Table 2:** Crude and adjusted odds ratios for outcome variables based on migratory status of households and individuals.

**Outcome Variable**	**Migratory Status**	**Crude**		**Adjusted**	
		**OR (95% CI)**	***p*** **-value**	**OR (95% CI)**	***p*** **-value**
**Based on household data**					
Financial barrier^a, c^ (outcome 1)	Host [REF]	1		1	
	Refugee	**3.28 (2.302, 4.66)**	**<0.001**	**3.10 (2.14, 4.49)**	**<0.001**
Pregnant women enrolled in ante-natal care program ^a, c^ (outcome 2)	Host [REF]	1		1	
	Refugee	**2.29 (1.16, 4.52)**	**0.017**	**2.26 (1.03, 4.65)**	**0.026**
Pregnant and/or lactating women enrolled in nutrition program ^a, c^ (outcome 3)	Host [REF]	1		1	
	Refugee	**18.82 (9.93, 35.68)**	**<0.001**	**20.99 (10.64, 41.41)**	**<0.001**
Received CLEAR COVID-19 awareness Info on accessing healthcare ^a, c^ (outcome 4)	Host [REF]	1		1	
	Refugee	**1.73 (1.18, 2.54)**	**0.005**	**1.75 (1.17, 2.63)**	**0.006**
**Based on individual data**					
Accessing proper healthcare ^b, d^ (outcome 5)	Host [REF]	**1**		**1**	
	Refugee	**3.27 (2.43, 4.41)**	**<0.001**	**3.10 (2.19, 4.39)**	**<0.001**

OR, Odds Ratio; CI, Confidence intervals. Significant results are shown in bold. ^a^Adjusted for sex of head of HH, highest education of HH, presence of disabled person in HH and HH size. ^b^Adjusted for sex, age, and employment status of individual. ^c^Generalized Linear Mixed Model. ^d^Binary Logistic regression. Significant results are shown in bold.

Refugee HHs were found to be significantly more likely to encounter financial barriers (Outcome 1) compared to the host HHs with the AOR of 3.10 (95% CI: 2.14, 4.49; *p* = <0.001).

Refugee HHs showed higher odds compared to host HHs for enrollment in ANC programs (Outcome 2) for pregnant women (AOR of 2.26, 95% CI: 1.03, 4.65; *p* = 0.026).

A similar pattern was seen in the enrollment of PLW in nutrition programs (Outcome 3) with refugee HHs having higher odds of enrollment with an AOR of 20.99 (95% CI: 10.64, 41.41; *p* = <0.001) in comparison to host HHs.

Refugee HHs were more likely to receive clear COVID-19 awareness information (Outcome 4) regarding healthcare access than host HHs, as exhibited by an AOR of 1.75 (95% CI: 1.17, 2.63; *p* = 0.006).

The random effect for sub-district (Upazila) variance was not significant across any of the analysis at HH level, suggesting that financial barrier, enrollment in ANC and nutritional program and receiving clear COVID-19 awareness info did not vary significantly across sub-districts. Given the minimal contribution of the random effect, this suggests that sub-district-level clustering had a negligible impact on the variability of the outcome.

Refugees were significantly more likely to access proper healthcare (Outcome 5) compared to hosts with an AOR of 3.10 (95% CI: 2.19, 4.39; *p* = <0.001).

## 4 Discussion

This study aimed to investigate whether differences in healthcare access exist between Rohingya refugees and the host community in Cox's Bazar, Bangladesh. The findings show differences in healthcare access between these two groups. Although the results showed that, compared to the host population, the refugees were likely to face more financial barriers, they had higher odds of enrolling in ANC and nutrition programs, received clearer COVID-19 awareness information and had a higher likelihood of accessing proper healthcare than the host population.

Financial barriers for refugees may indicate the challenges presented by out-of-pocket payments for accessing healthcare, even in organized camp settings such as the ones in Cox's bazaar. In line with the findings, many studies have highlighted financial barrier as one of the major barriers for refugees to access healthcare in their host country (28, 54), though the extent of these barriers can vary depending on the host country's legal and economic context. Despite the availability of subsidized and/or free healthcare for refugees (55), refugees may have to pay out-of-pocket when they need to seek care outside the camps, such as at private clinics or the district hospital, where they usually have to pay for consultations or they may face indirect costs related to healthcare access, such as transportation or purchasing medications (56). All these can exacerbate financial strain, especially when employment opportunities are limited (57, 58), such as in case of Rohingyas (20). The difference in financial barrier can also be attributed to several factors related to the host such as hosts being native to the region, often having better access to established healthcare networks, relatively more stable economic situations, or are more likely to be integrated into social support systems, which might mitigate the financial burden of healthcare (59).

The results also showed that more refugee pregnant women were enrolled in the ANC program compared to the hosts. These results can be explained by considering several factors. Despite the availability of ANC programs, the host population in impoverished areas may not be accessing these services as much as refugees. This could be due to the combination of more intensive outreach, logistical support, and targeted service delivery by humanitarian organizations operating in refugee camps, which often results in better physical access and awareness among refugees. These services are frequently situated within or near the camps and are designed to reach the refugee population consistently (35, 39). Given that refugees experience greater financial barriers (as shown in the results), they are also more likely to rely on free or subsidized healthcare services offered through these humanitarian programs, according to previous literatures (60, 61). Therefore, the higher enrollment of refugees in ANC services may reflect the effectiveness of these targeted and cost-free service delivery models.

The higher proportions of refugee PLW enrollment in the nutrition program found in the results of this study can be attributed to the focused nutritional support provided by various aid agencies, which often prioritize nutritional interventions for refugee populations due to their greater risk of malnutrition (62, 63), however, while not including the needs of the host population (64). Studies in Bangladesh showed that the prevalence of anemia was 49% among pregnant women (65) and approximately two in every three women of reproductive age in Bangladesh had been reported as underweight (66). This shows that while refugees have vulnerability (67, 68), host communities may face similar conditions but are not actively included in these interventions (34, 64).

Because of their living and environmental circumstances, the Rohingya refugees in Bangladesh were expected to be more susceptible to COVID-19 (69). The higher proportion of refugees receiving clear awareness information on COVID-19 could be linked to the extensive presence of international and local organizations in the camps with targeted health communication efforts aiming to curb the spread of the virus within vulnerable populations (69, 70). These efforts likely included tailored messaging and more intensive outreach in refugee camps where refugees are more easily reached due to their concentrated living conditions (71, 72) compared to host population whose living conditions are more dispersed over a greater area (73).

Although the results showed that refugees were more susceptible to financial barrier while accessing healthcare, the analysis of individual data reveals that refugees were significantly more likely to access proper healthcare compared to the host community. Several factors may contribute to this disparity. Firstly, as explained above, targeted humanitarian assistance plays a crucial role. Usually, NGOs and international organizations specifically design and provide proper healthcare services, contrary to the availability of poor-quality pluralistic health services in the host community, to meet the needs of refugees (39), often offering these services for free or at highly subsidized rates. These targeted interventions may increase the likelihood of refugees accessing proper healthcare (10, 74). Some studies (75–77) have discussed such equity concerns, pointing to disparities in healthcare access with regard to host communities in areas impacted by refugee populations. Given that host communities also have access to camp-based services in addition to the non-camp based health services, further research is needed to understand why they continue to report lower proportion of healthcare utilization.

Secondly, healthcare services seem to be strategically located within or near refugee camps which eliminates physical barriers such as long travel distances, lack of transport, or security checkpoints for refugees (55, 78). These services are often integrated into the camp's infrastructure, making them more accessible and easier to reach for the refugee population (55, 79). In contrast, host community members may face logistical challenges in accessing these clinics, especially if they are located within or adjacent to refugee camps (31, 74). The physical distance and potential transportation issues could possibly deter hosts from utilizing these services. Moreover, the higher population density within the refugee camps may also account for the greater proportion of refugees accessing the healthcare services, as the spatial concentration of both the population and available health facilities likely facilitates higher proportion of refugees accessing services (80).

Perceived barriers may also play a significant role. Host community members might feel reluctant to seek care at facilities primarily serving refugees, perceiving these clinics as less suitable or services being of lower quality or stigmatized as “refugee healthcare,” leading to lower utilization rates among host HHs, even when the services are available to them (54, 81, 82). Social acceptance can translate into assumptions about the quality of care provided at facilities that cater to refugees, reinforcing the stigma and reluctance to access these services. This reluctance can lead them to rely more on pharmacies, drug shops, or traditional healers, which are often more familiar and perceived as more culturally appropriate, even though they are categorized as improper healthcare in this context (83–85). Moreover, refugees often face restrictions on their movement in and out of the camp, which might limit their ability to access government or private clinics located outside the camps. These restrictions may make them more reliant on the healthcare services provided within the camp, which are likely to offer proper health services, further increasing their utilization of NGO clinics (79, 86).

We also observed that greater proportion of unemployed individuals accessed proper healthcare compared to employed. This raises the possibility that unemployment might offer individuals more flexibility and time to attend medical appointments, especially if their healthcare is free or highly subsidized. This result is in line with a study which showed unemployment was associated with positive health-seeking behavior (87). Exploring this relationship further could provide valuable insights.

While the host community may have more financial resources overall to access healthcare within the national health system, refugees can benefit from the financial support provided by humanitarian organizations (88, 89), primarily to access free or subsidized proper healthcare services provided within the camps. However, the same may not be true for refugees that are not residing in a camp setting (90). Additionally, the provision of healthcare services in refugee camps is not same across countries (91). Hence the generalization of results should be done with great caution. We acknowledge that the quantitative results alone do not fully explain the relationship between financial barriers and higher healthcare access to health services among refugees. Thus, qualitative studies are planned to be conducted as part of the broader research project.

The larger project aims at exploring the differences in healthcare access between refugees and host communities and how these differences are experienced and addressed, using mixed methods and from different perspectives. The results from the current quantitative study will be further explored, in another study, using a qualitative approach by interviewing refugees, host community and healthcare providers in Cox's Bazar. This is expected to provide deeper insights into the multiple factors underlying the disparities observed in this analysis.

### 4.1 Strengths and limitations

This study benefits from the availability of a unique dataset that includes both refugee and host community households, surveyed using the same questionnaire. This design enabled direct comparisons of healthcare access disparities across the two groups. During sampling, care was taken to ensure adequate representation of both communities, including attention to the proportional inclusion of female-headed households.

One of the limitations is that the data were collected in 2020, during the COVID-19 pandemic and may not fully reflect healthcare access patterns under non-pandemic situations. However, similar patterns in healthcare access barriers have been documented in non-pandemic contexts, suggesting that the results are not solely influenced by the pandemic. For example, studies conducted before and after 2020 in other displacement settings report persistent challenges in healthcare accessibility, especially in fragile and resource-constrained environments. Although the data are not recent, the underlying issues remain highly relevant (46, 92, 93). Healthcare access challenges for both refugees and host populations continue to be a concern in humanitarian contexts, including Bangladesh. Targeted interventions within refugee camps may help mitigate some of the access challenges refugees face (93), but this does not imply they receive better or equitable access overall. It is important to note that the findings show that both refugees and host communities experience barriers to healthcare, though the nature and extent differ.

Another limitation is the study's cross-sectional design, which restricts the ability to infer causal relationships. Additionally, the analysis was bound by the available variables in the dataset. The analysis was constrained by the reliance on available variables because the data was secondary. Conducting phone interviews due to COVID-19 restrictions introduced limitations, including reduced depth of responses due to the absence of personal interaction. While efforts were made to adjust for potential confounders, there remains the possibility of residual confounding due to unmeasured or inadequately measured variables, which could influence the observed associations. Preliminary data checks revealed that some variables, such as marital status and age, were unreliable and had to be excluded from the final analysis, potentially impacting the study's comprehensiveness. The limited sample size for some analyses, such as those involving PLW in HHs, could also have led to potential type II errors.

Lastly, the outcome variables do not fully capture the multidimensions of healthcare access. It should also be noted that access to healthcare does not guarantee better health outcomes. For this, the healthcare provided has to be need based and of good quality (94). This study has not assessed the type of healthcare sought (e.g., primary vs, secondary vs. emergency), quality of the healthcare provided or health outcomes, and results must be interpreted with caution. The findings of this study are specific to the context of Cox's Bazar, Bangladesh, and may not be generalizable to other refugee settings with different socio-political, economic or living conditions as well as non-camp settings.

## 5 Conclusion

This study reveals significant differences in healthcare access between Rohingya refugees and host communities in Cox's Bazar, Bangladesh. While refugees experienced more financial barriers to accessing healthcare compared to their hosts, they benefit from higher enrollment in ANC and nutrition programs, access to proper healthcare, and clearer COVID-19 awareness information. These differences can highlight the role of targeted humanitarian aid and the strategic location of healthcare facilities within refugee camps, which make some health services more accessible for refugees. However, focus on refugee health needs can inadvertently overlook the host communities, leading to feelings of exclusion, exacerbating inequalities, and potentially straining social cohesion, thus reinforcing the need for more inclusive approaches that address the needs of both communities. Future studies should evaluate the quality and outcomes of healthcare services to ensure that they meet the specific needs of each community. Additionally, future studies should also look into refugee healthcare access in camp and non-camp settings, as camps often provide centralized services, while in non-camp settings, services are more general and dispersed. This research offers valuable insights that can contribute to the development of a more equitable and inclusive healthcare system for all.

## Data Availability

Publicly available datasets were analyzed in this study. This data can be found here: https://microdata.unhcr.org.
